# Prenatal diagnosis of mosaic trisomy 2 and literature review

**DOI:** 10.1186/s13039-020-00504-3

**Published:** 2020-08-25

**Authors:** Ting Wang, Jufei Lian, Congmian Ren, Huamei Huang, Yanlin Huang, Ling Xu, Laiping Zheng, Chanhui Cai, Li Guo

**Affiliations:** Medical Genetic Center, Guangdong Women and Children Hospital, Guangzhou,Chi531 Xinnan Road, Panyu District, Guangzhou, China

**Keywords:** Non-invasive prenatal testing, Trisomy 2, Mosaicism, Single nucleotide polymorphism array, Fluorescence in situ hybridization, Chromosomal abnormality, Prenatal diagnosis

## Abstract

**Background:**

We presented two cases of mosaic trisomy 2 with high risk of maternal serum screening and non-invasive prenatal testing (NIPT). The invasive amniocentesis was performed and genetic tests including karyotype, single nucleotide polymorphism array(SNP-array), interphase fluorescence in situ hybridization (FISH) were employed to detect the chromosomal abnormality.

**Results:**

Cytogentic analysis of the case 1 and 2 showed a mosaic karyotype consisting of two cell lines (mos 47,XY,+2[8]/46,XY[19] and mos 47,XX,+2[7]/46,XX[28], respectively). SNP-array using DNA extracted from uncultured amniotic fluid cells revealed a result of arr[GRCh38](2)x2~3, which indicated that chromosome 2 may be trisomy of mosaicism in both two cases. The results of interphases FISH confirmation test showed that three red signals of the CEP 2 specific probe in 14%(14/100) and 12%(12/100) of the two cases’ cells, respectively, which indicated a mosaicism for trisomy 2 in the uncultured amniocytes. Fetal ultrasound of case 1 suggested that the long bone is smaller than the gestational age, while the case 2 showed that the biparietal diameter (BPD), head circumference (HC) and femur length (FL) were smaller than gestational age along with abnormal cardiac structure.

**Conclusions:**

We presented two cases with mosaic trisomy 2 and performed confirmatory genetic testing using cultured and uncultured amniocytes. When maternal serum screening and NIPT suggesting high risk, genetic counselor should be alert for increasing possibility of chromosomal anomalies if combined with abnormal ultrasound findings.

## Background

Complete trisomy 2 is a lethal chromosomal abnormality, accounting for 1% to 5–6% in early pregnancy and 1.1% in all spontaneous abortions [1]. It is estimated that the prevalence of trisomy 2 mosaicism in chorionic villi sampling (CVS) is about 1/2000 ([2–4] (Sifakis)), compared with about 1/58000 in amniocentesis during the second trimester ([5] (Sago)). In addition, the frequency of pseudomosaicism in trisomy 2 was the highest among all chromosomes in the karyotype analysis of amniotic fluid cell culture ([6, 7] (Hsu)). However, case reports of true fetal mosaicism of trisomy 2 are extremely rare. Up to present, only 21 cases of mosaic trisomy 2 have been reported in the prenatal diagnosis via amniocentesis or CVS. The prenatal manifestations of fetus with true mosaic trisomy 2 are quite variable, mainly including intrauterine growth restriction (IUGR),cerebral ventriculomegaly, oligohydramnios, congenital diaphragmatic hernia, cleft palate, cardiac defects and may also be associated with abnormal maternal serum screening [8, 9].

In recent years, non-invasive prenatal testing (NIPT) has developed rapidly and been widely used in the prenatal screening initially of the main autosomal non mosaic trisomies and sex chromosogwme aneuploidies. NIPT can evaluate chromosomes other than 13, 18, 21, X and Y. Rare autosomal trisomy (RAT) can have sometimes adverse effects on pregnancy outcomes [10, 11]. The most common RAT detected in NIPT involved chromosome 7 and 16 [12]. However, cases indicated high risk by NIPT for trisomy 2 are rare, and cases that have been confirmed to be true fetal mosaicism with chromosome 2 have not been reported.

In this paper, we described two prenatal cases with high risk of NIPT for trisomy 2 which were diagnosed by single nucleotide polymorphism array(SNP-array) and conventional karyotype analysis. The previously published literatures about mosaic trisomy 2 were reviewed, and the ultrasound findings and pregnancy outcomes were discussed.

## Methods

### Subjects

Case 1: A 39-year-old Chinese woman, gravida 3, para 1, abortion 1, was referred to our genetic center for counseling due to the advanced maternal age. Case 2: A 29-year-old Chinese woman, gravida 3, para 2, was referred to our center for genetic counseling because of the high risk of serum screening in second trimester. The first trimester ultrasound screening of case 1 was not available while the case 2’s showed unremarkable findings. The second trimester NIPT results of the two pregnant women both indicated that chromosome 2 were increased. The two couples were nonconsanguineous and had no personal or family history of congenital anomaly. This study was approved by the Ethics Committee of Guangdong Women and Children Hospital and the informed consent was obtained from the two couples.

### Cytogenetic karyotype

The follow-up amniocentesis was performed in second trimester (case 1 in 19 weeks and case 2 in 23 weeks). 20 ml of amniotic fluid were collected and cultured by using in situ vessel. The preparations were conducted according to standard procedures (GTG-banding). Twenty metaphases from independent colonies were counted, five of which were karyotyped. If mosaicism was encountered, all the metaphases that can be obtained from two independent culture vessels are analyzed. The International System for Human Cytogenomic Nomenclature (ISCN 2016) was employed to describe the karyotypes [13].

### SNP-array

10 ml of amniotic fluid were collected for SNP-array. The DNA were extracted from uncultured amniocytes using QIAamp DNA Blood Mini Kit (QIAGEN, Germany) according the recommended procedure. NANODROP 2000(Thermo, USA) was employed to test the DNA concentration. SNP-array analysis was performed using CytoScan 750 K chip (Affymetrix, USA). ChAS software was used to interpret the chip data.

### Fluorescence in situ hybridization (FISH)

Interphase FISH on uncultured amniocytes was employed to confirm the diagnosis of the mosaic trisomy 2 using chromosome 2 centromeric probe (Abbott Vysis CEP Spectrum Red probe, USA) and chromosome 22 BCR probe (Abbott Vysis CEP Spectrum Green probe, USA) as control. The experiment was performed according to the standard FISH protocol.

## Results

The maternal serum screening of case 1 were not available while the results of case 2 revealed a Down syndrome risk of 1/576 calculated from the levels of 1.67,3.11,0.83,0.98 and 0.63 multiples of the median (MoM) for a-fetoprotein (AFP), β-human chorionic gonadotropin(β-hCG), unconjugated estriol (uE3), pregnancy-associated plasma protein-A (PAPP-A) and nuchal translucency (NT), respectively. The NIPT results of two cases both indicated high risk of trisomy 2.

**Fig. 1:**
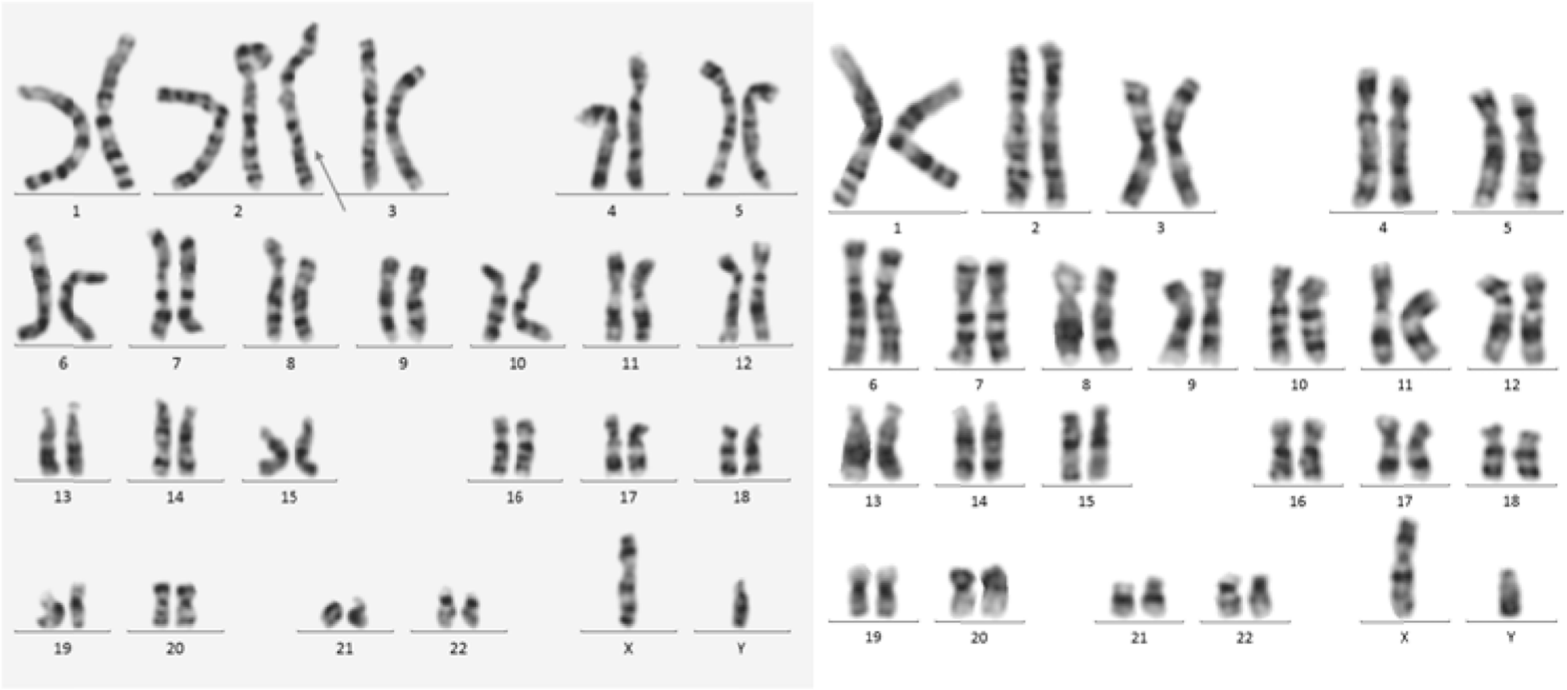
Karyotypes of case 1, The arrow shows the extra chromosome 2

**Fig. 2:**
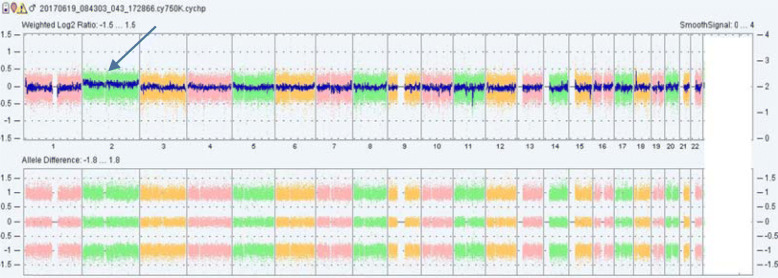
Single nucleotide polymorphism array of case 1 using DNA extracted from uncultured amniotic fluid cells. The arrow shows the whole genome view of chromosome 2 slightly deviated from baseline and it indicates that chromosome 2 may be trisomy of mosaicism

**Fig. 3:**
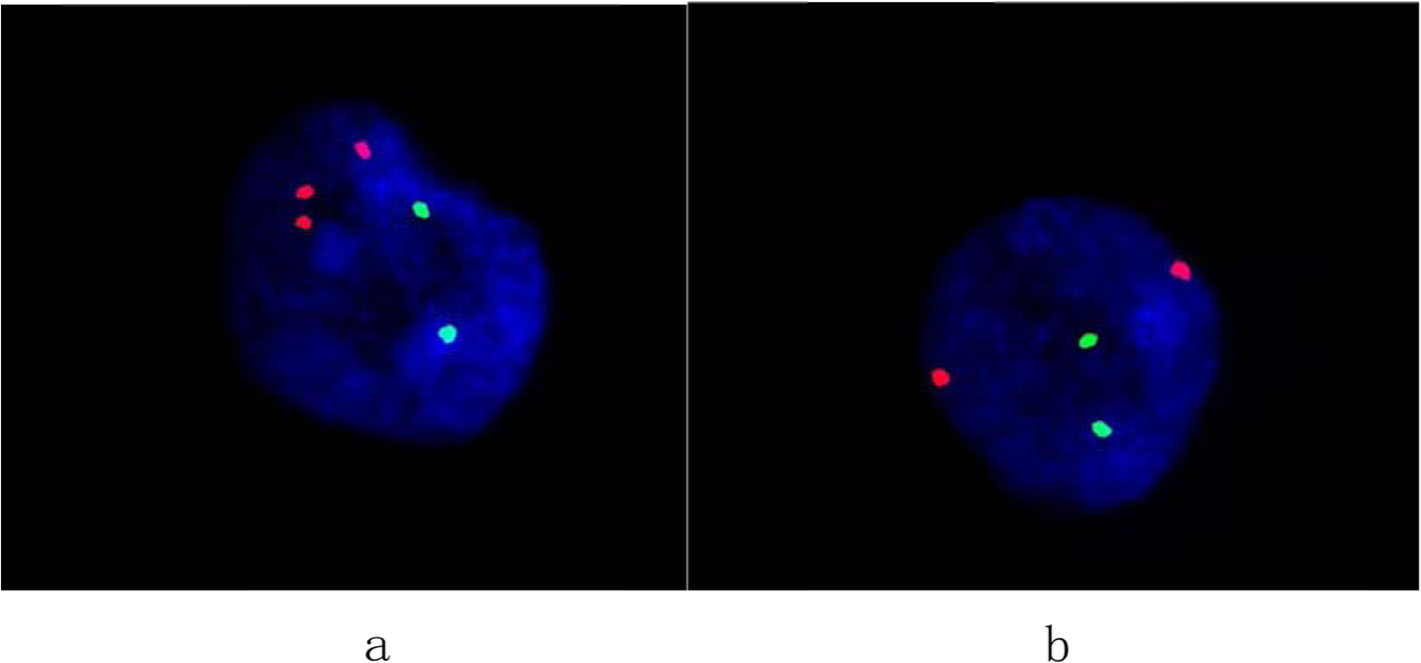
Interphase FISH on uncultured amniocytes of case 1 using Vysis chromosome 2 centromere probe (spectrum red) and chromosome 22 BCR probe (spectrum green) as control. **a**: Three red signals and two green signals indicated a cell with trisomy 2. **b**: Two red and green signals indicated a cell with disomy 2. The result confirmed that both cases were mosaic aneuploidies

For case 1, cytogentic analysis showed a karyotype of mos 47,XY,+2[8]/46,XY[19] as shown in Fig. 1. Of 27 colonies cultured from amniotic fluid cells,8 colonies had the karyotype of 47,XY,+2 and the other 19 colonies had the karyotype of 46,XY.The abnormal cell line were obtained from two independent in situ vessels and 3 metaphases from culture A,5 metaphases from culture B. For case 2,the result revealed a karyotype of mos 47,XX,+2[7]/46,XX[28] as shown in Fig. 1. Three of the abnormal metaphases were from culture A and four were from culture B. The mosaic percentage in cultured amniocytes of two cases was 29.6%(8/27) and 20%(7/35), respectively. According to the guidelines of prenatal mosaicism, these findings were interpreted as level III mosaicism [7]. SNP-array using DNA extracted from uncultured amniotic fluid cells revealed a result of arr[GRCh38](2)×2~3 as shown in Fig. 2 which indicated that chromosome 2 may be trisomy of mosaicism in both two cases. The mosaic percentage of two cases deduced from the chip analysis was about 25 and 22%, respectively. The results of interphases FISH confirmation test showed that three red signals of the CEP 2 specific probe in 14%(14/100) and 12%(12/100) of the two cases’ cells, respectively, which indicated a mosaicism for trisomy 2 in the uncultured amniocytes (Fig. 3).

**Fig. 4:**
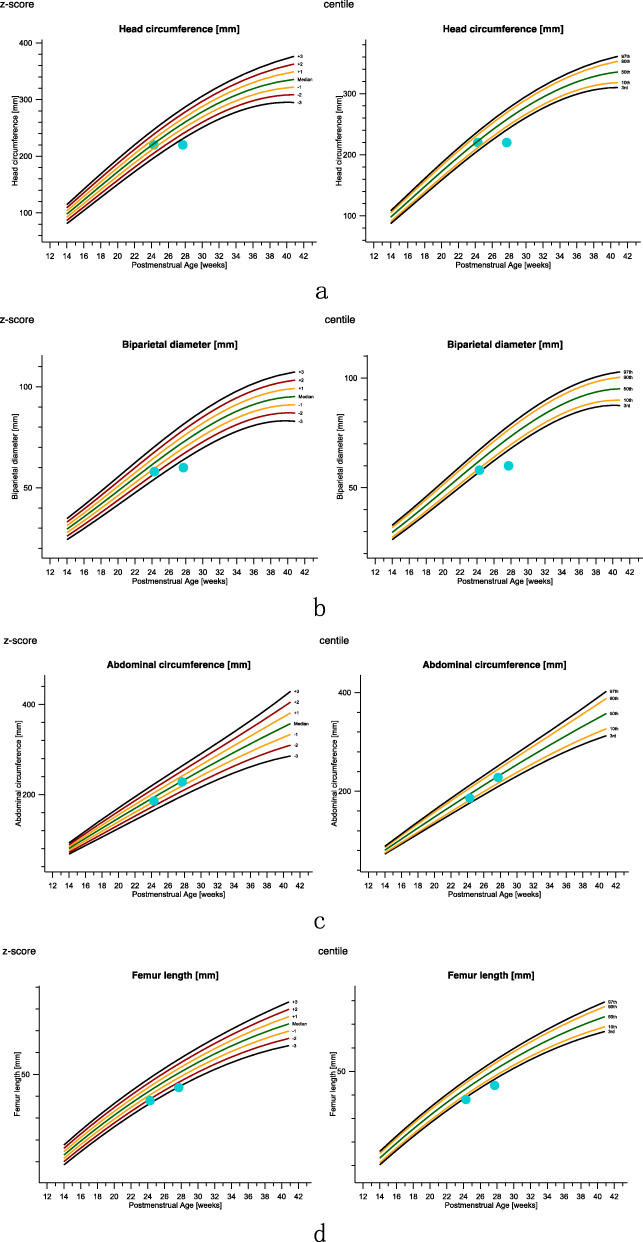
Fetal biometry charts of fetal growth based on INTERGROWTH -21st International Standards for Fetal Growth (v1.6.4). Biparietal diameter, head circumference and femur length measurements of case 2(right dots) were smaller than gestational ages which indicated the fetal was IUGR. The femur length measurements of case 1(left dots) was smaller than gestational ages. **a**: Z-scores and centile of head circumference. **b**: Z-scores and centile of biparietal diameter. **c**: Z-scores and centile of abdominal circumference. **d**: Z-scores and centile of femur length

The subsequent detailed ultrasound was recommended in second trimester for the screening of malformations. Fetal ultrasound of case 1 in 24^+ 2^ weeks of gestations suggested that all the long bones is smaller than the gestational age. For case 2, ultrasound in 27^+ 5^ weeks of gestations showed that the biparietal diameter (BPD), head circumference (HC) and femur length (FL) were smaller than gestational age along with abnormal cardiac structure, which indicated the fetal was IUGR. The Z-scores and centile of fetal growth parameters in two cases with mosaic trisomy 2 were documented in Table 1 and Fig. 4 according to International Standards for Fetal Growth (v1.6.4) [14].

**Table 1:** Z-scores and centile of two fetuses with mosaic trisomy 2

		Z-scores				centile			
	GA	HC	BPD	AC	FL	HC	BPD	AC	FL
Case 1	24^+ 2^	−0.27	−1.60	− 0.86	− 2.36	39.5	5.43	19.6	0.9
Case 2	27^+ 5^	−4.23	− 4.33	−0.21	−3.15	0.00	0.00	41.6	0.08

*GA* gestational age, *BPD* biparietal diameter, *HC* head circumference, *AC* abdominal circumference, *FL* femur length

Both pregnant women of case 1 and case 2 decided to terminate their pregnancies due to the abnormal ultrasound findings and molecular cytogenetic results. The term for the termination of pregnancy was in 29 and 32 weeks, respectively. Both couples declined to further pathological fetal examination.

## Discussion

True fetal mosaicism (TFM) of trisomy 2 is extremely rare in the prenatal diagnosis. The present cases of our study provide evidence for the use of multiple genetic detections on uncultured amniocytes to rapidly confirm the existence of low level mosaic trisomy 2 in amniocentesis. Previous published literatures suggest that follow-up amniocentesis should be performed for confirmation in case mosaic trisomy 2 was encountered in cytogenetic analysis of chorionic villi in prenatal diagnosis [5, 15]. Our approach of level III mosaicism is to combine the results of molecular cytogenetic detection and ultrasound findings for comprehensive evaluation of pregnancy outcome, and to confirm by uncultured amniocytes FISH. We consider that if a sample is independently tested by three different methods and the same result is obtained, the result should be reliable. For the two cases, trisomy 2 was found in both uncultured and cultured amniotic fluid cells. This means that the two fetuses may be true mosaicism of trisomy 2.

The clinical phenotypes of fetuses with mosaic trisomy 2 are diverse, including more frequently but not limited to microcephaly, IUGR, cleft lip, scoliosis, congenital diaphragmatic hernia, cardiac defects, growth and motor delay, caudal dysgenesis. The summary of trisomy 2 mosaicism in previous published literature and present cases was shown in Table 2. So far, at least 23 cases of mosaic trisomy 2 have been detected by amniocentesis in addition to our cases. Chen CP et al. [23] reported a male preponderance in fetus with mosaic trisomy 2 and a natural selection against female conceptuses based on the sex ratio (1.8,11 males/6 females). However, it can be seen from Table 2 that the sex ratio of fetal mosaic trisomy 2 is 1.3 (12 males and 9 females), which indicates that the quantitative gap between the genders is narrowing, meaning there may be no difference between the genders in fetuses with mosaic trisomy 2. Harrison et al. [8] presented a pregnant woman with an elevated maternal serum human chorionic gonadotrophin (MShCG) level of 3.67 MoM, which gave birth a fetus with mosaic trisomy 2 and maternal uniparental disomy 2. High level of MShCG was also found in the present case 2, indicating a association between mosaic trisomy 2 and abnormal maternal serum screening [1, 5, 8, 9, 15, 17, 19, 20, 23].

**Table 2:** The summary of trisomy 2 mosaicism in published literature and present cases

Reference	Karyotype	Indication	Phenotype	Outcome	Trisomy 2 mosaic rate in amnioce-ntesis	Molecular findings
Case1	47,XY,+2/46,XY	AMA, Abnormal NIPT and ultrasound	Long bones smaller than gestational weeks	TOP	29.6%	SNP-array:arr[GRCh38] (2)×2~3; Interphase Fish: 14% trisomy 2
Case2	47,XX,+2/46,XX	Elevated MShCG, Abnormal NIPT and ultrasound	Cardiac defects, IUGR	TOP	20%	SNP-array:arr[GRCh38] (2)×2~3; Interphase Fish: 12% trisomy 2
Sago et al. [5]	47,XY,+2/46,XY	Elevated MSAFP,IUGR	Hypotonia, microcephaly, growth retardation, developmental delay, prominent occiput, beaked-prominent nose, flat malar area, thin lip, pointed chin, pectus excavatum, inguinal hernias, V- shaped palate, rocker- bottom feet, congenital heart defects, hydronephrosis, vesicoureteral reflux, delay myelination, a thin corpus callosum, hippocampal dysplasia, portal fibrosis	Delivery at 30 week,1135 g	22.60%	NA
Casey et al. [16]	47,XY,+2/46,XY	Ventriculomegaly, extremity discordance	Posteriorly rotated ears, high arched palate, widely spaced nipples, lowset bilateral simian creases, bilateral overlapping 4th and 5th fingers	Delivery at 39 week, 2445 g	12%	NA
Webb et al. [17]	47,XX,+2/46,XX	AMA,oligohydramnios, Elevated MSAFP,IUGR	Renal failure, vesicouteric reflux, patent ductus ateriosus, congenital pyloric stenosis, hiatus hernia	Delivery at 31 week, 765 g	23.40%	maternal UPD(2)
Harrison et al. [8]	47,XY,+2/46,XY	Elevated MShCG, normal AFAFP, oligohydramnios, IUGR, breech presentation	Growth failure, hypothyroidism, hyaline membrane disease, bronchopulmonary dysplasia	Delivery at 36 week, 1710 g	32.50%	maternal UPD(2)
Cramer et al. [18]	NA	IUGR, breech presentation	Hypertelorism, midface hypoplasia, frontal bossing, unilateral proptosis, contralateral ptosis, broad halluces, radial deviation of the wrist, scoliosis, unilateral radioulnar hypoplasia, gross motor and growth delay (Pfeiffer syndrome like)	Livebirth	NA	NA
Robinson et al. [19]	47,XY,+2/46,XY	Abnormal maternal serum screen for AFP and hCG, Down risk 1/90	Mild dysmorphic features, absent gall bladder, cystic left kidney, 13th left rib, Mild unilateral talipes	TOP	56.70%	multiple tissue mosaicism (Interphase FISH)
Pappas et al. [20]	47,XY,+2/46,XY	AMA,Abnormal ultrasound ventriculomegaly Elevated AFAFP	Lumbosacral spina bifida	TOP	23.40%	NA
Sifakis et al. [4]	47,XY,+2/46,XY	AMA	No phenotypic abnormalities	TOP	16%	NA
Sifakis et al. [21]	NA	IUGR, oligohydramnios	coarctation of the aorta	TOP	1.90%	NA
Hsu et al. [15]	47,XY,+2/46,XY	AMA	NA	Livebirth	4%	NA
	47,XX,+2/46,XX	Elevated MSAFP	No phenotypic abnormalities	Stillbirth	6.30%	NA
	47,XX,+2/46,XX	NA	NA	IUFD	6.70%	NA
	47,XX,+2/46,XX	Elevated MSAFP, oligohydramnios	Abnormal abortus, dolichocephaly	TOP	33.30%	NA
	47,XX,+2/46,XX	NA	NA	Stillbirth	20%	NA
Chen et al. [9]	47,XY,+2/46,XY	Abnormal MSS, Down risk 1/12,IUGR,severe oligohydramnios, ventricular septal defect	Micrognathia, depressed nasal bridge, low-set ears, and preaxial polydactyly of the right hand	TOP	26%	aCGH:242.9 Mb duplication of 2p25.3-q37.3;Interphase Fish: 11.1% trisomy 2
Chen et al. [22]	47,XX,+2/46,XX	AMA	No phenotypic abnormalities	Livebirth	5%	Interphase Fish: 3.4% trisomy 2
Chen et al. [23]	47,XY,+2/46,XY	Abnormal MSS	No phenotypic abnormalities	Livebirth	4.80%	Interphase Fish: 16% trisomy 2
Chen et al. [24]	47,XX,+2/46,XX	AMA,IUGR	Low-set ears, macroglossia, clenched hands	TOP	30%	aCGH:trisomy 2 mosaicism; Interphase Fish: 12% trisomy 2
Tuğ et al. [1]	47,XX,+2/46,XX	AMA,Abnormal MSS, Down risk:1/50	Cardiac dextroposition and diaphragmatic hernia	TOP	14%	NA
Bui et al. [25]	47,XY,+2/46,XY	NA	Transversal hemimelia	TOP	NA	NA
	47,XY,+2/46,XY	NA	Ambiguous external genitalia	TOP	NA	NA

*NA* not available, *AMA* advanced maternal age, *MSAFP* maternal serum α-fetoprotein, *IUFD* intrauterine fetal death, *IUGR* intrauterine growth restriction, *MShCG* maternal serum human chorionic gonadotrophin, *MSS* maternal serum screen, *TOP* termination of pregnancy, *aCGH* array comparative genomic hybridization, *UPD* uniparental disomy, *SNP-array* single nucleotide polymorphism array, *AFAFP* amniotic fluid α-fetoprotein, *FISH* fluorescence in situ hybridization

American College of Medical Genetics and Genomics (ACMG) issued guidelines in 2016 that recommended NIPT screening for all pregnant women, mainly including aneuploidy on chromosome 21, 18 and 13, but not for other chromosomal abnormalities [26]. However, other chromosomal abnormalities are usually detected concurrently due to the high throughput of NIPT. Benn et al. demonstrated that the relative frequency of trisomy 2 was 3.4% in a total of 499 RATs identified by cfDNA analysis [27]. Wan et al. identified 2 cases of trisomy 2 in 59 cases with high risk of RAT detected by NIPT and one of the positive case turned out to be a homozygosity of chromosome 2 by vertification of CMA [11]. To our knowledge, our study is the first presentation of decribing two cases of high risk for chromosome 2 in the NIPT detection and to make confirmatory genetic testing for mosaic trisomy 2.

Case 1 and 2 showed 29.6 and 20% mosaic ratio of trisomy 2 in karyotype analysis, while 14 and 12% in uncultured amniocytes FISH, respectively. Different mosaic ratio between cultured amniocytes (karyotype) and uncultured amniocytes (FISH) may be due to the selective growth of the different cell lineages in the culture process [28]. A bias of the selection to a particular cell type was presented during the culture whereas FISH and SNP-array performed with uncultured samples from different cell lineages do not have such potential bias [29]. As can be seen from Table 2, majority of the mosaic trisomy 2 cases performed with molecular test have discordant mosaic ratio of cytogenetics and molecular detection. FISH on uncultured amniocytes is a practical approach to detect low level of mosaicism, which can also distinguish true fetal mosaicism from pseudomosaicism [23]. Some authors [28, 29] considered that CMA is superior to standard cytogenetics in detecting mosaicism. In our opinion, FISH should be recommended as the preferred detection method for low level mosaicism, rather than CMA due to the limitations in detecting low level mosaicism trisomy [30].

Genetic counseling about fetal mosaicism is a challenge also including mosaic trisomy 2 on account of the unpredictable outcome. Even with the most accurate prenatal testing, true fetal mosaicism does not necessarily mean that individuals will have any phenotypic consequences, as the source or proportion of cells carrying abnormalities cannot be predicted [31]. When it comes to addressing the risk of mosaicism recurrence in a family, a detailed family history and all molecular tests must be considered, just as in a non-mosaic genetic disease [32]. If mosaicism is found in more than one generation and in the offspring of more than one generation, the most likely explanation for the recurrence of the disease is that the family is affected by chromosomal instability disorder, which needs to be confirmed by genetic testing [32]. When the results of prenatal samples (amniotic fluid, chorionic villi, cord blood) were discordant, the results of amniotic fluid are more reliable, because the cells in amniotic fluid come from multiple germ layers and are more representative of the true condition of the fetus. Mosaic trisomy 2 is a rare chromosomal anomaly with wide phenotypic spectrum, and its severity may be related to the rates of mosaicism. Detailed ultrasound examination is helpful to determine the prognosis of mosaic trisomy 2.

The formation of mosaicism may involve two cell division error, including non-disjunction during mitosis and postzygotic correction of aneuploidy during meiosis [7]. The first situation is the major mechanism that causes mosaicism [33]. The rescue mechanism of the second situation would lead to UPD which was defined by the presence of a chromosome pair from only parent [34]. Chromosome 6,7,14,11,15 were confirmed to contain imprinted gene associated with clinical phenotypes while the phenotypic effects of imprinted regions on chromosome 2,16,20 were unclear [35].

The fetal cell-free DNA was derived from the cytotrophoblasts of placenta. Therefore, a positive NIPT result may indicate that the placenta contains abnormal cell line. Confined placenta moscaisim (CPM) was defined as the presence of abnormal cells only in placenta. In this study, the positive NIPT result and amniocentesis for mosaic trisomy 2 indicated that the two fetuses may be TFM.

In a recent review, the ontogenetic and pathogenetic views on somatic chromosomal mosaicism were demonstrated [36]. The author believed that chromosomal mosaicism may mediate genomic/ chromosomal instability and intercellular diversity in a bottleneck fashion, as chromosomal mosaicism has the ability of dynamic changes during ontogeny. However, the existence of small cell populations with abnormal karyotypes led to difficulties in interpretation and detection. In the post-genomic era, it is possible to identify molecular and cellular pathways of chromosomal mosaicism using advanced genome-wide scanning techniques and bioinformatics tools.

In summary, we presented two cases with mosaic trisomy 2 and performed confirmatory genetic testing using cultured and uncultured amniocytes. When maternal serum screening and NIPT suggesting high risk, genetic counselor should be alert for increasing possibility of chromosomal anomalies if combined with abnormal ultrasound findings.

## Data Availability

The datasets used and/or analyzed during the current study are available from the corresponding author on reasonable request.

## Abbreviations

NIPT: Non-invasive prenatal testing
AC: Abdominal circumference
BPD: Biparietal diameter
FISH: Fluorescence in situ hybridization
FL: Femur length
HC: Head circumference
SNP-array: Single nucleotide polymorphisms array
CVS: Chorionic villi sampling
IUGR: Intrauterine growth restriction
RAT: Rare Autosomal trisomy
MoM: Multiples of the median
β-hCG: β-human chorionic gonadotropin
MShCG: Maternal serum human chorionic gonadotrophin
AFP: a-fetoprotein
uE3: unconjugated estriol
PAPP-A: pregnancy-associated plasma protein-A
NT: nuchal translucency
CMA: chromosomal microarray analysis
TFM: true fetal mosaicism
CPM: confined placental mosaicism.
UPD: uniparental disomy
